# Sex Differences in Newly Diagnosed Severe Aortic Stenosis in British Columbia (B.C.)

**DOI:** 10.3390/diseases13070191

**Published:** 2025-06-22

**Authors:** Aishwarya Roshan, Jeffrey Yim, Shamikh Lakhani, Jennifer Wang, Aamiya Sidhu, Eric C. Sayre, Karin Humphries, Janarthanan Sathananthan, David Wood, Michael Y. C. Tsang, Darwin F. Yeung, Christina Luong, Parvathy Nair, Kenneth Gin, John Jue, John G. Webb, Teresa S. M. Tsang

**Affiliations:** 1 Department of Medicine, University of British Columbia, Vancouver, BC V6T 1Z4, Canada; aishwi@gmail.com (A.R.);; 2 Vancouver General Hospital Artificial Intelligence Echocardiography Core Laboratory, Vancouver, BC V5Z 1M9, Canada; 3 Division of Cardiology, University of British Columbia, Vancouver, BC V6T 1Z4, Canada

**Keywords:** sex differences, aortic stenosis, transcatheter aortic valve replacement, cardiac surgery, cardiac outcomes

## Abstract

Background: Despite its high prevalence, little is known about the effect of sex on the management and outcomes of aortic stenosis (AS). We sought to characterize the effect of sex on the clinical evaluation for and provision of aortic valve replacement (AVR), including surgical (SAVR) and transcatheter aortic valve replacement (TAVR), and the subsequent morbidity and mortality outcomes. Methods: A comprehensive chart review was conducted on all patients with a first diagnosis of severe aortic stenosis (AS) at Vancouver General and University of British Columbia hospitals from 2012 to 2022. Exact chi-square and Kruskal–Wallis tests were used to evaluate the variables of interest. Results: A total of 1794 studies met the inclusion criteria, comprising 782 females (44%) and 1012 males (56%). Females were significantly older than males at the time of the first diagnosis (79 versus 75 years, *p* < 0.001). Females were significantly less likely to be evaluated by the TAVR clinic or cardiac surgeon or to receive aortic valve intervention (*p*-value ≤ 0.001). Females were significantly more likely to be rejected for TAVR due to older age (OR 0.23 (0.07, 0.59)), comorbid conditions (OR 0.68 (0.47, 0.97)), and frailty (OR 0.23 (0.07, 0.59)). Females were significantly more likely to be rejected for SAVR on the basis of frailty (OR 0.66 (0.46, 0.94)). Females also had significantly higher rates of 1-year mortality, hospitalization, and heart failure hospitalization compared to males (*p*-values < 0.05). Conclusions: Our data suggest significant sex-based discrepancies in the management of AS. Females with severe AS are diagnosed later in life and are less likely to be evaluated for valve intervention. They are less likely to receive intervention due to older age, frailty, and multimorbid conditions. Further research is warranted for a more effective identification and follow up of aortic stenosis, as well as timely referral for AVR, where appropriate, especially for females.

## 1. Introduction

Aortic stenosis (AS) is a common and progressive valvular heart disease worldwide with significant morbidity and mortality [1]. It is well-established in the previous literature that severe aortic stenosis has a high rate of mortality if left untreated [2]. Over the past several years, transcatheter aortic valve replacement (TAVR) has gained traction globally as a safe and effective alternative to surgical aortic valve replacement (SAVR) for patients deemed non-optimal candidates for the latter. TAVR has been shown to provide morbidity and mortality benefits for both women and men [2,3,4,5]. Data on the impact of sex on the practice patterns of AS management and outcomes remains unclear [6]. Vancouver is one of Canada’s pioneering centers for TAVR innovation and implantation. We sought to characterize the effect of sex on specialist referrals; the clinical evaluation for and provision of aortic valve replacement (AVR), including SAVR and TAVR; reasons for non-referral for intervention; and outcomes. We sought to understand the impact of sex on the management of AS to facilitate the improvement of service delivery and cardiovascular outcomes.

## 2. Materials and Methods

### 2.1. Study Design and Selection

The study procedure and protocols were designed in accordance with the Declaration of Helsinki and received approval from the University of British Columbia institutional review board. A retrospective chart review was conducted for all patients with a new diagnosis of severe AS between 1 January 2012 and 31 December 2022, at a tertiary care center. Consecutive transthoracic echocardiograms (echo) meeting severe aortic stenosis criteria were obtained from Syngo (version 20), a secure, password-protected imaging archiving system hosting echocardiograms from both facilities between 1 January 2012 and 31 December 2022. Echocardiograms demonstrating severe aortic stenosis were identified using the criterion defined in the 2020 ACC/AHA Guideline for the Management of Patients with Valvular Heart Disease. This included echocardiograms with an aortic maximum velocity greater than or equal to 4 m/s or a mean pressure gradient greater than or equal to 40 mm Hg and an aortic valve area less than or equal 1.0 cm^2^. Echocardiograms identifying low-flow, low-gradient aortic stenosis and paradoxical low-flow, low-gradient aortic stenosis were also included [1].

Clinical data were abstracted from local electronic medical records. Variables of interest were abstracted including age, sex, and the presence or absence of coronary artery disease, dyslipidemia, previous myocardial infarction, previous heart failure, atrial fibrillation, ischemic stroke, hypertension, chronic kidney disease, permanent pacemaker, peripheral vascular disease, or chronic obstructive pulmonary disease. Symptoms at echocardiogram diagnosis were quantified using the New York Heart Association (NYHA) classification scale for heart failure as well as the Canadian Cardiovascular Society (CCS) angina scoring systems. Patients were classified based on whether they were hospitalized during the time of diagnosis or diagnosed as an outpatient. Decisions surrounding eligibility for SAVR or TAVI were made by a multidisciplinary heart health team. This team consisted of interventional cardiologists, general cardiologists, cardiac surgeons, nurse practitioners, general practitioners, and allied health professionals including physiotherapists, occupational therapists, and social workers. Patients and family members were informed of the risks and benefits of undergoing and not undergoing aortic stenosis intervention prior to personal decision making.

### 2.2. Clinical Endpoints

Data were collected on the dates of the initial cardiology visit, TAVR consultation referral, TAVR consultation, surgical consultation referral, and surgical consultation and the date of AVR (either TAVR or SAVR). Wait times were calculated, including the time from diagnosis to TAVR/surgical consultation visit, time from diagnosis to AVR, and time from TAVR/SAVR consultation referral to AVR. One-year major adverse cardiovascular outcomes, defined as the development of stroke, persistent atrial fibrillation or flutter, all-cause hospitalization, heart failure hospitalization, or all-cause mortality within one year of the echocardiographic diagnosis of AS, were abstracted from electronic medical records. Reasons for non-referral to both TAVR and surgical consultation, as well as reasons for not receiving TAVR or SAVR, were analyzed and compared between males and females.

### 2.3. Statistical Analysis

Descriptive statistics were computed for the demographic, baseline, and clinical characteristics of patients. Continuous variables were expressed as the median with the interquartile range, while categorical variables were expressed as the number of events with percentages. Comparisons of demographic, baseline, clinical, and echocardiographic characteristics were made using exact chi-square tests for categorical variables or Kruskal–Wallis tests for continuous data (e.g., time to visit for specialist visits). Analyses were performed using SAS version 9.4 (SAS Institute Inc., Cary, NC, USA). A *p*-value of <0.05 was considered to be statistically significant. A univariate model analysis was conducted to delineate patient characteristics predictive of receiving TAVI. Variables in the univariate model included demographic data (age, sex, and rurality) and patients’ past medical history, as listed in Table 1. Independent predictors of receiving TAVI from this univariate analysis included advanced age, CKD, female sex, high symptom burden (having NYHA III or IV symptoms and/or CCS 2+ symptoms), and prior stroke. These characteristics were subsequently analyzed in a multivariable analysis.

## 3. Results

Over a 10-year period (2012–2022), 1794 patients [782 females (44%) and 1012 males (56%)] were identified to have severe AS on echo for the first time. Patient characteristics are summarized in Table 1. Females were significantly older than males at the time of the first diagnosis (79 versus 75, *p* < 0.001). Males were significantly more likely to have been diagnosed with cardiometabolic comorbidities, including dyslipidemia (57.6% versus 48.1%, *p*-value < 0.001), coronary artery disease (55.1% versus 47.6%, *p*-value 0.002), and diabetes (27.7% versus 23.5%, *p*-value 0.049), whereas females were significantly more likely to have hypertension (74.2% versus 69.1, *p*-value 0.020).

Whether a patient was assessed by a specialist (cardiologist, TAVR clinic, and/or a surgeon) and/or received intervention (TAVR or SAVR) was tabulated and compared between sexes (Table 2). Males were significantly more likely to be evaluated by a surgeon for the consideration of SAVR (57.1% versus 47.1%, *p*-value < 0.001); more likely to be evaluated by either a surgeon or the TAVR clinic for the consideration of TAVR or SAVR, respectively (75.5% versus 68.3%, *p*-value 0.001); and more likely to receive AVR (65.8% versus 52.4%, *p*-value < 0.001). There was no difference in the median wait times between females and males for specialist evaluation and the receipt of intervention (Table 3).

Along with these differences in specialist care and intervention between the sexes, females were found to have higher rates of 1-year hospitalization and 1-year heart failure hospitalization (Table 4). All findings were highly significant, with all *p*-values being < 0.015 and most being < 0.001.

The reasons for non-referral to specialist evaluation and not receiving intervention were also examined. Clinician reports on each patient were analyzed by researchers to identify reasons for patient non-referral and/or the non-receipt of intervention. While a patient may have had more than one reason for not being referred or receiving TAVR and/or SAVR, the largest contributing factor, as delineated by the surgeon/interventionalist on their consultation report, was indicated in our analysis. In determining characteristics such as frailty, STS score, or cognitive function, clinicians relied heavily on standardized, objective measures in combination with clinical judgment.

Females were significantly more likely to not be referred to a TAVR clinic for the consideration of TAVR due to older age (4% versus 1%, *p*-value 0.013) and being asymptomatic (12% versus 8%, *p*-value 0.027). Being asymptomatic was defined as a lack of symptoms at rest or with exertion as expressed by the patient during their clinic visit. Males were more likely to not be referred to a TAVR clinic for requiring concurrent cardiac surgery (29% versus 24%, *p*-value < 0.001) (Table 5). There were no significant differences between males and females in non-referral to a SAVR clinic (Table 6).

Females were significantly more likely to be rejected for TAVR on the basis of older age (4% versus 1%, *p*-value = 0.001), multiple comorbid conditions (13% versus 9%, *p*-value = 0.031), frailty (5% versus 2%, *p*-value = 0.004), having poor TAVR anatomy, and having multivalvular heart disease (5% versus 2%, *p*-value 0.004). Males, on the other hand, were rejected for TAVR on the basis of requiring another concurrent cardiac surgery (30% versus 15%, *p*-value < 0.001), as well as having a low Society of Thoracic Surgeons (STS) score (21% versus 17%, *p*-value = 0.063) (Table 7).

Females were also significantly more likely to be rejected for SAVR on the basis of frailty (15% versus 10%, *p*-value = 0.019) (Table 8).

A multivariable analysis was conducted to evaluate independent predictors of receiving TAVR. Amongst those receiving AVR, females were more likely to receive TAVR compared to their male counterparts [OR of 1.47 (1.07,2.01); *p* = 0.016] in the multivariate analysis.

## 4. Discussion

Our data suggests that significant sex differences existed across our large cohort in the management of severe AS. Specifically, males had a higher comorbidity burden compared to females. They were more likely to have dyslipidemia, coronary artery disease, and diabetes, as well as requiring a statin and anti-platelet agent. This is consistent with prior research that suggests that males have more cardiovascular comorbidities including myocardial infarction, stable angina, ischemic stroke, peripheral arterial disease, heart failure, and cardiac arrest [7,8].

However, despite the higher burden of cardiovascular disease amongst males, the literature points to females having higher rates of morbidity and mortality post-acute coronary syndrome diagnosis, particularly within ST elevation myocardial infarctions [9,10,11]. In our study, females were more likely to require hospitalization for heart failure and for other reasons, when compared to males within 1 year of their AS diagnosis.

The diagnosis and treatment of female patients at a more advanced age have long been observed in the context of acute coronary syndrome (ACS) and are the leading explanation for the mortality differences observed between females and males [10]. Females are less likely to undergo diagnostic procedures, such as coronary angiography; less likely to receive treatments, such as percutaneous coronary intervention, coronary artery bypass graft, or optimal medical therapy; and more likely to have prehospital delays in obtaining adequate care for their ACS [10,11,12,13,14,15,16]. Likewise, our study showed that females were less likely to be evaluated by the TAVR clinic or a surgeon and less likely to receive AVR, which may explain the differences in morbidity between the sexes.

Females were significantly less likely to be referred for and/or receive TAVR due to old age. This held the strongest statistical significance out of all reasons. While echocardiographic parameters were not directly analyzed in our paper, our finding of reduced female patient referral coupled with prior research delineating differences in LV structure, function, and hemodynamics between the sexes underlines the importance of developing sex-specific criteria in defining AS severity [17]. The next most important reason for non-intervention in females was frailty. Even amongst individuals who received surgical assessment, females were significantly more likely to be rejected for SAVR on the basis of frailty. The assessment of frailty by clinicians was made through various objective and validated clinical tools including Dalhousie Clinical Frailty Scale and the Edmonton Frail Scale. While clinical judgment played a role, it was informed by standardized frameworks to reduce bias. Both SAVR and TAVR confer significant mortality and morbidity benefits, and thus, understanding the reasons for the lack of treatment is pivotal in enabling physicians to provide optimal care. Both age and frailty are implicated in the lack of intervention in female patients. One of the highest predictors of frailty is advanced age [18]. In our cohort, females were diagnosed with severe AS at a later age, when compared to males. It is unclear whether they developed symptoms earlier, as well. In light of these findings, the consideration of sex-based triaging criteria for intervention for the improvement of outcomes could be considered. One of the most significant reasons for not pursuing SAVR within our cohort was patient preference, which is also identified within the literature amongst female patients [19,20,21,22].

It is prudent to acknowledge the broader social determinants of health, such as access to primary care, socioeconomic status, access to food and shelter, and caregiving support, that greatly contribute to the sex-based differences observed in our study. Females are disproportionately disadvantaged by such social inequities, which inevitably leads to the observed differences in referral patterns, intervention receipt, and cardiovascular outcomes [6]. Future prospective studies examining sex-based differences should assess the influence of socioeconomic factors on disease management and outcomes as a critical research objective.

Despite being a large study, one limitation of our study includes the fact that it was conducted within a single institution with a single electronic medical record. This project therefore does not account for hospitalizations, diagnoses, and events obtained outside of the Vancouver Coastal Health jurisdiction and assumed that the patients of our study only obtained care within this health authority. As part of our future research prospects, we hope to validate our findings within multiple centers in Canada and North America. Moreover, the current study did not analyze whether patients were using SGLT2 inhibitors. The majority of years included in this retrospective review were before the introduction of SLGT2 inhibitors as cardiac medications.

## 5. Conclusions

There appeared to be significant sex-based discrepancies in the management of AS. Females were diagnosed with severe AS later in life. They were less likely to be evaluated for valve replacement (both TAVR and SAVR), and upon evaluation, they were also less likely to receive this intervention, due to their older age, frailty, and multimorbid conditions. Greater efforts are needed to identify females with severe AS earlier in disease progression. Further research is required to determine whether the lack of referral and receipt of AVR amongst females is an independent predictor of their significantly higher morbidity and mortality outcomes and whether triaging criteria for intervention incorporating sex will improve outcomes for female patients.

## Figures and Tables

**Table 1:** Baseline characteristics (demographics, past medical history, medications) of cohort stratified by sex.

Variables	Females	Males	*p*-Value
Demographic			
Age	78.94 ± 11.31	75.12 ± 11.21	<0.001
Sex	782 (43.6%)	1012 (56.4%)	-
Rurality	72/777 (9.3%)	129/1002(12.9%)	0.019
Hospitalized at Time of Diagnosis	222/779 (28.5%)	276/1006 (7.4%)	0.632
Past Medical History			
NYHA III/IV	264/775 (34.1%)	333/1005 (33.1%)	0.686
CCS 1+	151/775 (19.5%)	236/1005 (23.5%)	0.049
Dyslipidemia	372/774 (48.1%)	579/1006 (57.6%)	<0.001
Hypertension	575/775 (74.2%)	695/1006 (69.1%)	0.020
Coronary Artery Disease	369/775 (47.6%)	555/1007 (55.1%)	0.002
Previous Myocardial Infarction	92/774 (11.9%)	133/1007 (13.2%)	0.429
Previous Stroke	125/774 (16.2%)	152/1006 (15.1%)	0.553
Previous Heart Failure	197/774 (25.5%)	229/1005 (22.8%)	0.198
Peripheral Vascular Disease	83/774 (10.7%)	134/1007 (13.3%)	0.108
Chronic Obstructive Pulmonary Disorder	85/774 (11.0%)	112/1006 (11.1%)	0.939
Chronic Kidney Disease	164/774 (21.2%)	215/1006 (21.4%)	0.953
Diabetes	182/774 (23.5%)	279/1006 (27.7%)	0.049
Atrial Fibrillation	227/774 (29.3%)	263/1006 (26.1%)	0.148
Permanent Pacemaker	47/774 (6.1%)	48/1006 (4.8%)	0.243
Medications			
Beta-Blocker	292/762 (38.3%)	393/990 (39.7%)	0.587
ACE Inhibitor	227/762 (29.8%)	344/990 (34.8%)	0.031
Angiotensin Receptor Block (ARB)	151/762 (19.8%)	131/990 (13.2%)	<0.001
Calcium Channel Blocker	214/761 (28.1%)	238/988 (24.1%)	0.061
Mineralocorticoid Receptor Antagonist	31/762 (4.1%)	28/990 (2.8%)	0.181
Diuretic	291/761 (38.2%)	333/991 (33.6%)	0.050
Digoxin	35/762 (4.6%)	28/990 (2.8%)	0.053
Statin	316/762 (41.5%)	546/990 (55.2%)	<0.001
Anti-Platelet	261/762 (34.3%)	441/991 (44.5%)	<0.001
Oral Anticoagulant	179/762 (23.5%)	203/990 (20.5%)	0.145

**Table 2:** Specialist assessment and cardiac intervention stratified by sex.

Cardiac Consultations	Sex of Patient			
	Females	Males	*p*-Value	Odds Ratios (95% CI)
Seen by Cardiologist	676/781 (86.6%)	879/1009 (87.1%)	0.778	1.05 (0.788, 1.397)
Seen by TAVR Clinic	403/777 (51.9%)	501/1003 (50.0%)	0.444	0.926 (0.764, 1.122)
Seen by Surgeon	368/781 (47.1%)	575/1007 (57.1%)	<0.001	1.494 (1.232, 1.811)
Seen by TAVR or Surgeon	534/782 (68.3%)	763/1010 (75.5%)	0.001	1.435 (1.159, 1.776)
Received AVR	408/779 (52.4%)	662/1006 (65.8%)	<0.001	1.75 (1.438, 2.129)
*Type of AVR:*				
None	371/779 (47.6%)	344/1006 (34.2%)	<0.001	None
TAVR	226/779 (29.0%)	293/1006 (29.1%)		
SAVR	182/779 (23.4%)	369/1006 (36.7%)		

**Table 3:** Wait times stratified by sex.

Time Intervals (Days)	Median Wait Times		
	Females	Males	*p*-Value
Time from Echo to Cardiologist Visit	28 (8–124)	29 (7–112)	0.762
Time from Echo to TAVR Clinic Visit	185 (75–593)	200 (62–621)	0.491
Time from Referral to TAVR Clinic Visit	38 (15–79)	35 (15–70)	0.429
Time from Echo to Surgical Consultation	203 (93–481)	174 (83–496)	0.439
Time from Referral to Surgical Consultation	55 (29–83)	51 (24–78)	0.141
Time from Echo to TAVR	378 (202–836)	339 (195–823)	0.223
Time from Echo to SAVR	307 (178–649)	354 (186–748)	0.435
Time from Echo to AVR	355 (196–750)	344 (190–768)	0.591

**Table 4:** Cardiovascular outcomes stratified by sex.

Cardiac Outcomes	Sex of Patient			
	Females	Males	*p*-Value	Odds Ratio (95% CI)
1-Year Mortality	127/779 (16%)	157/1006 (16%)	0.696	0.949 (0.73, 1.237)
1-Year Hospitalization	327 (42%)	359 (36%)	0.007	0.768 (0.631, 0.935)
1-Year HF Hospitalization	174/777 (22%)	176/1004 (18%)	0.012	0.737 (0.579, 0.938)
1-Year Persistent Atrial Fibrillation	59/777 (8%)	69/1004 (7%)	0.58	0.898 (0.616, 1.312)
1-Year Stroke	29/778 (4%)	27/1004 (3%)	0.221	0.714 (0.403, 1.261)

**Table 5:** Reasons for non-referral to TAVR clinic.

Reason	Frequency (*n*)	Female	Male	*p*-Value
Age	20	14/376 (4%)	6/509 (1%)	0.013
Multiple comorbid conditions	92	47/376 (13%)	45/509 (9%)	0.094
Frailty	33	19/376 (5%)	14/509 (3%)	0.105
Limited life expectancy	23	12/376 (3%)	11/509 (2%)	0.395
Patient decision	96	43/376 (11%)	53/509 (10%)	0.662
Concurrent cardiac surgery required	191	45/376 (24%)	146/509 (29%)	<0.001
Other	46	21/376 (6%)	25/509 (5%)	0.760
Low STS score	168	67/376 (18%)	101/509 (20%)	0.488
Poor TAVR anatomy	2	2/376 (1%)	0/509 (0%)	0.180
Asymptomatic	83	45/376 (12%)	38/509 (8%)	0.027
Cognitive dysfunction	27	15/376 (4%)	12/509 (2%)	0.172
Patient passed prior	42	16/376 (4%)	26/509 (5%)	0.633
Multivalvular disease	23	15/376 (4%)	8/509 (2%)	0.032
Poor functional status	10	5/376 (1%)	5/509 (1%)	0.751
Lost to follow up	29	10/376 (3%)	19/509 (4%)	0.447

**Table 6:** Reasons for non-referral to surgical evaluation.

Reason	Frequency (*n*)	Female	Male	*p*-Value
Age	67	34/413 (8%)	33/432 (8%)	0.800
Multiple comorbid conditions	235	110/413 (27%)	125/432 (29%)	0.490
Frailty	97	54/413 (13%)	43/432 (10%)	0.132
Limited life expectancy	26	13/413 (3%)	13/432 (3%)	1.000
Patient decision	120	57/413 (14%)	63/432 (15%)	0.768
Other	46	21/413 (5%)	25/432 (6%)	0.762
Poor surgical anatomy	13	8/413 (2%)	5/432 (1%)	0.411
Asymptomatic	85	45/413 (11%)	40/432 (9%)	0.493
Cognitive dysfunction	34	17/413 (4%)	17/432 (4%)	1.000
Patient passed prior	39	15/413 (4%)	24/432 (6%)	0.194
Multivalvular disease	1	0/413 (0%)	1/432 (0%)	1.000
Too high risk for surgery	16	10/413 (2%)	6/432 (1%)	0.319
Poor functional status	12	6/413 (2%)	6/432 (1%)	1.000

**Table 7:** Reasons for not receiving TAVR.

Reason	Frequency (*n*)	Female	Male	*p*-Value
Age	26	20/555 (4%)	6/712 (1%)	0.001
Multiple comorbid conditions	141	74/555 (13%)	67/712 (9%)	0.031
Frailty	46	30/555 (5%)	16/712 (2%)	0.004
Limited life expectancy	31	16/555 (3%)	15/712 (2%)	0.464
Patient decision	120	55/555 (10%)	65/712 (9%)	0.699
Concurrent cardiac surgery required	298	83/555 (15%)	215/712 (30%)	<0.001
Other	48	22/555 (4%)	26/712 (4%)	0.882
Low STS score	247	95/555 (17%)	152/712 (21%)	0.063
Poor TAVR anatomy	32	21/555 (4%)	11/712 (2%)	0.032
Asymptomatic	93	49/555 (9%)	44/712 (6%)	0.082
Cognitive dysfunction	31	17/555 (3%)	14/712 (2%)	0.271
Patient passed prior	71	31/555 (6%)	40/712 (6%)	1.000
Multivalvular disease	37	25/555 (5%)	12/712 (2%)	0.004
Poor functional status	15	7/555 (1%)	8/712 (1%)	1.000
Lost to follow up	31	10/555 (2%)	21/712 (3%)	0.205

**Table 8:** Reasons for not receiving SAVR.

Reason	Frequency (*n*)	Female	Male	*p*-Value
Age	91	44/594 (7%)	47/639 (7%)	1.000
Multiple comorbid conditions	377	179/594 (30%)	198/639 (31%)	0.757
Frailty	152	87/594 (15%)	65/639 (10%)	0.019
Limited life expectancy	30	14/594 (2%)	16/639 (3%)	1.000
Patient decision not to proceed	153	72/594 (12%)	81/639 (13%)	0.796
Other	47	21/594 (4%)	26/639 (4%)	0.658
Poor surgical anatomy	42	22/594 (4%)	20/639 (3%)	0.639
Asymptomatic	91	48/594 (8%)	43/639 (7%)	0.385
Cognitive dysfunction	43	21/594 (4%)	22/639 (3%)	1.000
Patient passed prior	47	17/594 (3%)	30/639 (5%)	0.103
Multivalvular disease	1	0/594 (0%)	1/639 (0%)	1.000
Too high risk for surgery	64	28/594 (5%)	36/639 (6%)	0.521
Poor functional status	33	16/594 (3%)	17/639 (3%)	1.000
Lost to follow up	31	10/594 (2%)	21/639 (3%)	0.100
Unclear	31	15/594 (3%)	16/639 (3%)	1.000

## Data Availability

The data presented in this study are available on request from the corresponding author.
